# Wild Life in a Hospital Barrack-Square

**Published:** 1914-04-11

**Authors:** 


### RELAXATIONS AND HOBBIES.

### Wild Life in a Hospital Barrack-Square.

Barrack-squares seldom are associated with the interests of wild life. Exceptions can be found in the drill-grounds of Tidworth. "Before the soldiers came"—this is the local anno Domini—much of the land on which the barracks stand grew crops of corn and had upon it farmhouses, thatched cottages, and barns; around was, and is now, open down, the park, and extensive woodlands. Where now is a town with some eight thousand inhabitants there was but a sprinkling of farmers and farm-hands. Vast as is the difference between then and now it has altered little the wild life of the place. The comings and goings of men and horses, the rattle of arms, the blare of the bugles, and the sounding of the trumpets have not scared the birds from their haunts; they have accepted the situation and have adapted themselves to the new environment.

Within three hundred yards of Jellalabad Barracks rooks nest in the branches and jackdaws in the hollows of old trees in the park as if no change had come. But, sad to say, the presence of the troops has demoralised them. Especially is this the case in summer-time, for then they collect about the camps pitched on the plain and are semi-parasitic on man. They become scavengers, foragers for trifles, bold thieves, low fellows consorting with crows, and they carry the manners of the camp back to the squares. Both rooks and jackdaws frequent the lines, search the manure-heaps for scraps, and squabble and fight over their finds. The jackdaws, alert, cunning, and more active than the rooks, slip in and out the scrimmages, avoid actual conflict with the heavier birds, and seem to get away with the larger share of the spoils.

Starlings also do not disdain such scraps as come their way. They frequently are foraging on the manure-heaps with the rooks, but as a rule they are intent on more natural food and run about the grass-plots by the squares searching for insects and the small snails that form the main of their food. So confident are they that even when squads of recruits march up and down within a few feet of them they do not fly away. The old farm buildings, in the thatch of which were the homes of their ancestors, have been razed; but they have adapted themselves to the times and make free of the new buildings for their nests. Whilst the starlings are busy with their nesting they run about the grass-plots in pairs, but at the beginning of June the young begin to come out and increase daily in numbers. The food supply in barracks then is no longer sufficient, so young and old fare forth to the downs and collect in flocks that contain many hundreds of birds. It is very pleasing to watch these assemblages skirmish for their food across the downs. The birds run along the grass, all moving in the same direction, with eager eyes searching the herbage, making little darts of the beak at insects and snails. Since the foremost take the cream of the supplies the rearmost grow dissatisfied with their position and rise and fly over the flock to take their turn in the van. But scarcely have they tasted the plenty before them than the new rear-rank, dissatisfied in its turn, is up and taking the lead. Thus there is incessant changing of position from rear to front. In this fashion the flocks work all day across large stretches of the plain. Just when the cherries are ripe they fly to the orchards and tax the gardens for the good they do the fields during the remainder of the year.

Swallows and martins hawk their food across the barrack-squares in numbers that increase as summer draws to its end. They skim over the fatigue-parties that cut the grass, and swoop round the men's heads to seize insects disturbed from their lurking-places by the scythes. They use his Majesty's forces as beaters for their sport; for they assemble and hang upon the flanks and rear of parties that march out across the plain, and make heavy bags of the winged creatures driven from the grass by the trampling feet of men and horses.

That strange bird the swift, so like the swallow tribe that it is commonly supposed to belong to it, lives in the air above the barrack-squares. Truly lives in the air, since, except for sleep and to sit upon two white eggs in a scanty nest beneath the eaves of some one of the thatched cottages in the old village, the swifts never leave the air. On the wing they catch their food, collect their nesting materials, drink their drink, buffet their rivals, court their wives, and consummate their marriages. At terrific speed they rush through the air, chasing in courtship, rivalry, or sport, uttering the weird screechings which give them the name of screech-devils, and used to cause some superstitious fear. As they fly, sometimes they may be observed suddenly to cease to use their wings, to fall rapidly for many feet, and as suddenly to right themselves and continue their career. They never stand upon the ground of their own will, nor perch on trees. If one is caught in its nest and placed on the ground, it is with difficulty that it gets on wing again—perhaps because their legs and feet are so feeble that they cannot spring into the air to make the first wing-stroke. The great proportional length of their wings must add to the difficulty of getting under way. Their feet are like the feet of wood-peckers—all the toes turned to the front; thus they are eminently suited for clinging to the ledges of rocks and to the entrances of the holes in which they nest, but are nearly useless for locomotion.

Larks sing at all hours of daylight above the squares. They nest in the rough grass between the back of the officers' mess-blocks and the barracks. Any day in spring they may be seen in pairs demurely walking about the grass-plots feeding with the sparrows and the starlings. So tame are they that I have seen them feeding close to the feet of a man who was practising for the high-jump on the turf by the hospital block. It is pretty to see the cock bird at nesting-time. He runs excitedly round the hen, erects his crest, and warbles a few notes of his song as he goes.

Hedge-sparrows nest in the thick hedges which line the road that runs before the mess-blocks. They feed on the roads and in the gardens, and come daily to peck up small scraps dropped from the meat-carts that deliver at the back premises of the mess. They take scarcely any notice of passing troops, just slipping into the shelter of the bottom of the hedge and coming out again the moment the men have passed. In the same hedges chaffinches, greenfinches, and yellow-hammers nest regularly, undisturbed and almost unnoticed. After sunset owls flit out from the park and flap across the squares. Their screechings and hootings, and the crowing of the pheasants disturbed in their roosting-places in the park, are common sounds of the night.

Blackbirds, thrushes, warblers of several kinds, fly-catchers, corn-buntings, partridges, magpies—half the birds of the British Isles—can be watched and studied in amongst the barracks or within half a mile of this great range of new brick buildings. The recruit may learn his drill to the tune of the skylark, and divert his mind with the antics of a dozen varieties of birds so soon as he gets a "stand easy."

